# Disulfideptosis-associated lncRNAs reveal features of prognostic, immune escape, tumor mutation, and tumor malignant progression in renal clear cell carcinoma

**DOI:** 10.18632/aging.205534

**Published:** 2024-02-08

**Authors:** Xungang Li, Xinxi Deng, Taobin Liu, Wensheng Zhang, Jin Tao

**Affiliations:** 1 Department of Urology, Jiu Jiang No. 1 People’s Hospital, Jiujiang, Jiangxi 332000, P.R. China; 2 Department of Pediatric, Jiujiang University Affiliated Hospital, Jiujiang, Jiangxi 332000, P.R. China

**Keywords:** Disulfidptosis, lncRNA, immune escape, cell death pattern, renal clear cell carcinoma

## Abstract

Purpose: Investigating the role of lncRNAs associated with the latest cell death mode (Disulfideptosis) in renal clear cell carcinoma, as well as their correlation with tumor prognosis, immune escape, immune checkpoints, tumor mutational burden, and malignant tumor progression. Searching for potential biomarkers and targets for renal clear cell carcinoma.

Methods: Downloaded the expression profile data and clinical data of 533 cases of renal clear cell carcinoma from the TCGA database, and randomly divided them into a test set (267 cases) and a validation set (266 cases). Based on previous research, 13 genes associated with Disulfideptosis were obtained. Using R software, lncRNAs with a differential expression that is related to the prognosis of renal clear cell carcinoma and associated with Disulfideptosis were screened out. After univariate Cox regression analysis, Lasso regression analysis, and multivariate Cox regression analysis, lncRNAs with independent predictive ability were obtained. A predictive risk model was established based on the risk scores. Verification was carried out between the obtained high-risk and low-risk groups and their subgroups (including Age, Gender, tumor mutational burden (TMB), tumor grading, and staging). Subsequently, a nomogram was established, and a calibration curve was generated for verification. Performed GO (Gene Ontology) and KEGG (Kyoto Encyclopedia of Genes and Genomes) functional enrichment analyses. Downloaded the values of Tumor Immune Dysfunction and Exclusion (TIDE) for all samples and calculated the difference between the high and low-risk groups. Selected human renal tumor cell lines (786-O, OS-RC-2, A-498, ACHN) and human renal cortex proximal tubule epithelial cell line (HK-2). The RNA expression levels of the above lncRNAs in each cell line were analyzed using RT-qPCR (Real-time Quantitative PCR Detecting System). Used siRNA (small interfering RNA) to knock down FAM225B in 786-O and OS-RC-2 cell lines, and then performed *in vitro* cell experiments to validate the functional characteristics of FAM225B.

Results: Our constructed predictive model includes 5 lncRNAs with an independent predictive ability (FAM225B, ZNF503-AS1, SPINT1-AS1, WWC2-AS2, LINC01338), which can effectively distinguish between patients in high and low-risk groups and their subgroups. The 1, 3, and 5-year AUC (Area Under the ROC Curve) values of the established nomogram are 0.756, 0.752, and 0.781, respectively. The 5-year AUC value is higher compared to other clinical characteristics (Age: 0.598, Gender: 0.488, Grade: 0.680, Stage: 0.717). After the knockdown of FAM225B, the proliferation, migration, and invasion abilities of renal cancer cell lines OS-RC-2 and 786-O all decreased.

Conclusion: We have constructed and validated a prognostic model based on Disulfideptosis-associated lncRNAs. This model can effectively predict the high or low risk of patient prognosis and can distinguish the tumor cell mutational burden and immune escape capabilities among high-risk and low-risk patients. This predictive model can serve as an independent prognostic factor for renal clear cell carcinoma, providing a new direction for personalized treatment of patients with renal clear cell carcinoma.

## INTRODUCTION

Many cancer therapies are designed to kill cancer cells through apoptosis [1]. However, many cancer cells can find ways to evade treatment-induced apoptosis, ultimately leading to treatment resistance and disease recurrence [2]. Disulfideptosis is a newly discovered and identified form of cell death, which holds promise for exploring a new cancer treatment method based on this mechanism. This study indicates that when cells with high expression of the SLC7A11 protein experience glucose starvation, Disulfideptosis is triggered. In preclinical models, treatment with a glucose inhibitor can induce Disulfideptosis in cancer cells with high expression of SLC7A11, effectively suppressing tumor growth, and it has no significant toxicity to normal tissues [3]. The research team discovered in their 2020 study that certain cancer cells may be sensitive to glucose transporter inhibitors due to their high expression of SLC7A11 and the resultant “addiction” to extracellular glucose [4]. Overall, the main cause of Disulfideptosis is that the supply of NADPH cannot meet the cellular process of reducing cystine to cysteine, resulting in disulfide stress. This induces actin cytoskeleton protein disulfide bonding and cytoskeletal contraction, peeling from the plasma membrane, and ultimately leading to cell death. Insufficient cellular glucose intake and excessive cystine intake can both induce Disulfideptosis.

Because this is a newly discovered form of cell death, research related to it is currently a blank slate, especially when compared to previously discovered forms of cell death such as apoptosis, necroptosis, autophagy, ferroptosis, pyroptosis, and necrosis [5]. Disulfideptosis appears to be quite unique. As we know, apoptosis is usually associated with cell contraction [6], Necroptosis, on the other hand, involves cell swelling and leakage of cell contents [7], In contrast, Disulfideptosis is mainly associated with disulfide bonds on the cytoskeleton. The regulators and effectors of different cell death pathways remain very attractive therapeutic targets.

Kidney cancer has long been one of the top three tumors in men, especially renal clear cell carcinoma [8]. In 2022, there will be approximately 81,800 new cases of kidney cancer (52,360 in men and 29,440 in women) and 14,890 deaths (9,920 in men and 4,970 in women) in the United States [9]. Although its incidence has decreased [10], there is a long way to go in the prevention, diagnosis and treatment of kidney cancer [11]. Although Disulfideptosis has many potential specific mechanisms and available benefits, its other potential mechanisms and biomarker profiles for different types of cancers, especially renal clear cell carcinoma, remain unknown and it will be of great interest whether it can be used as a therapeutic target for renal clear cell carcinoma.

In conclusion, Disulfideptosis is a promising area for renal clear cell carcinoma research, and, lncRNAs associated with Disulfideptosis in renal clear cell carcinoma are currently unavailable. Therefore, we hope to explore whether lncRNAs associated with Disulfideptosis are related to prognosis, immune escape, tumor mutational load, immune checkpoint, and tumor malignant progression in renal clear cell carcinoma through this study, which is expected to make some contributions to further studies of Disulfideptosis.

## MATERIALS AND METHODS

We used five cell lines, four kidney cancer cell lines (786-O, OS-RC-2, A-498, ACHN), and one human renal cortical proximal tubular epithelial cell (HK-2), all from Procell (Wuhan, Hubei, China). Growth medium used: A-498, ACHN and HK-2 using MEM (with NEAA) + 10% FBS + 1% P/S, 786-O and OS-RC-2 using RPMI-1640 + 10% FBS + 1% P/S. All cells were grown at 37°C and 5% CO_2_. Freezing conditions were in liquid nitrogen. Cells were digested using 0.25% trypsin (Biosharp, Hefei, China) during passaging, and all cells above were digested for 1 min in a 37°C incubator.

### Acquisition of raw data

Clinical data of 533 renal clear cell carcinoma cases and expression profile data of all RNAs were downloaded from TCGA (The Cancer Genome Atlas). Similarly, 403 simple nucleotide variation data were downloaded from TCGA. Genes associated with Disulfideptosis from previous studies on Disulfideptosis [3], Tumor Immune Dysfunction and Exclusion (TIDE) data generated from the RNA transcriptome expression profiles of TCGA were downloaded from TIDE (http://tide.dfci.harvard.edu/) to calculate their TIDE values.

### Obtaining Disulfideptosis-related lncRNAs

Based on the downloaded TCGA RNA transcriptome expression profile, we used Perl software (bictype.perl) to separate the RNA transcriptome expression profile into lncRNA and mRNA expression profiles. Then, using R software (packages “ggplot” and “ggalluvial”), we depicted the co-expression relationship between lncRNA and Disulfideptosis-associated genes via Pearson correlation analysis (correlation with *p* < 0.05) as a Sankey diagram.

### Construction of the prognostic signature

Using R software (packages “survival”, “caret”, “glmnet”, “survminer”, and “timeROC”), we conducted univariate Cox regression analysis on Disulfideptosis-associated lncRNAs (significance criterion was *p* < 0.001), obtained Disulfideptosis-associated lncRNAs significantly associated with prognosis time, and plotted a forest map. Then we conducted a Lasso regression analysis (randomly dividing it into two groups, performing cross-validation, and finding the minimum error value). After that, we performed a multivariate Cox regression analysis of related factors, filtering out independent prognostic factors (5 disulfidptosis-related lncRNAs), thus constructing the prognostic signature. Using R software (packages “tidyverse”, “ggplot2”, “ggExtra”), we plotted a correlation heatmap of these 5 independent prognostic lncRNAs and 13 Disulfideptosis-associated genes through Pearson correlation analysis (correlation with *p* < 0.05).

### Evaluation and validation of the risk model

Using R software (packages “survival”, “caret”, and “survminer”), we randomly divided all 533 samples into two groups (training group and testing group). We then conducted a chi-square test on the clinical characteristics (Age, Gender, Grade, and Stage) of both groups, comparing whether there is a significant difference in the clinical characteristics of the two groups. A *p*-value less than 0.05 is considered statistically significant. Using R software (package “pheatmap”), we plotted risk curves, survival status maps, and risk heatmaps for both the training and testing groups (divided into high and low-risk groups based on the median). Using R software (packages “survival” and “survminer”), we plotted Kaplan-Meier (KM) curves for high and low-risk groups in both the training and testing sets, including Overall Survival (OS) and Progression Free Survival (PFS). Using R software (the “survival” package), we conducted univariate and multivariate independent prognostic analyses for the risk model. Using R software (packages “survival”, “survminer”, and “timeROC”), we generated the 1, 3, and 5-year ROC curves for the risk model and the 5-year ROC curves for Age, Gender, Grade, and Stage, and made a comparison. Using R software (packages “limma” and “scatterplot3d”), we performed a Principal Component Analysis (PCA) on the risk model to see if it can distinguish all lncRNAs, Disulfideptosis-associated genes lncRNAs, Disulfideptosis-associated genes, and all genes and visualized the results.

### Construction of the predictive nomogram

Using R software (packages “survival”, “regplot”, and “rms”), we incorporated the risk model into the construction of a predictive nomogram based on the risk score. Subsequently, we generated calibration curves for the 1, 3, and 5-year periods of the nomogram.

### Clinical characteristics of the risk model

Using R software (“survivor”, “survminer” package), survival curves were plotted for patients of different genders, tumor grades, and tumor stages based on the risk model to verify the applicability of our constructed risk model to patients of different clinical trait groups.

### Functional and pathway enrichment analysis

GO function enrichment analysis: Using R software (“colorspace”, “stringi”, “ggplot2”, “circlize”, “RColorBrewer”, and “ggpubr” packages), risk-differentiated Disulfideptosis-associated lncRNAs were analyzed for which functions they were enriched with and visualized, including cellular component (CC), molecular function (MF), and biological process (BP), presented as bar and circle charts. KEGG pathway enrichment analysis: Using R software (“colorspace”, “stringi”, “ggplot2”, “circlize”, and “RColorBrewer” packages), KEGG pathway enrichment analysis was performed for risk-differentiated Disulfideptosis-associated lncRNAs and visualized as bubble graphs and circle plots.

### Analysis of tumor-infiltrating immunocyte and immune checkpoints

GSVA analysis was performed using R software (“limma”, “GSVA”, “GSEABase”, “pheatmap”, and “reshape2” packages) on samples from high and low-risk groups, looking for correlations with immune function, and presented as heat maps.

### Tumor immune dysfunction and exclusion (TIDE) and estimation of tumor mutational burden (TMB)

Based on the expression profile of TCGA, TIDE data of all samples were downloaded from TIDE (http://tide.dfci.harvard.edu/), and TIDE values were calculated using R software (“limma”, “ggpubr” package) to calculate the TIDE values of samples from high and low-risk groups and compare them to assess the immune escape ability of different high and low-risk groups and visualize them as box plots.

Using the R software (“maftools” package), the TMBs in the high and low-risk groups were analyzed based on the simple nucleotide variation data downloaded from TCGA above, the differences were compared, and the different mutation types were displayed, and the 15 genes with the highest mutation frequencies were selected for display and visualized in waterfall plots. The relationship between different high and low TMB and patient survival time was analyzed using R software (“survival”, “survminer” package), and KM curves were generated. Also, the relationship between different high and low TMB in the high and low-risk groups and patient survival time was analyzed and KM curves were generated.

### RNA extraction and RT-qPCR

The RNA from cell lines and tissues was extracted using Trizol (Shanghai Kangwei Biotech, China), chloroform, isopropanol, 75% ethanol (Solarbio, Beijing, China), and RNase-free water. The extracted RNA was quantified, followed by reverse transcription into cDNA using a reverse transcription kit (Servicebio, Wuhan, China). Subsequently, real-time fluorescence quantitative PCR was performed using 2 × SYBR Green PCR Master Mix (Servicebio, Wuhan, China). The relative expression levels of the target genes were calculated using the 2^−ΔΔCt^ method. The experiments were repeated three times using different PCR runs, and the average values were obtained. β-actin was used as the internal reference gene.

### Primer sequences

β-actin F: CATGTACGTTGCTATCCAGGC R: CTCCTTAATGTCACGCACGAT. Human-FAM225B F: TCGGGATAGTGATGGCAAGC R: GCCACCTCTGTGGTCCTAAC. Human-LINC01338 F: GCCCAGGCTTCCCGATTATT R: ATGGGTTTGACCGTCGATGT. Human-SPINT1-AS1 F: CCGGGTACTTGAGCTCCCTA R: TGATCAGCCCGGGAGACTTT. Human-WWC2-AS2 F: GGGTCGTGTTTGCCCTTAGA R: GCCCTAAATGCGGTCAAAGC. Human-ZNF503-AS1 F: CATTCTCCACCCTGCCACAT R: AGGCATCTTGGCAGAAGGAC.

### Cell transfection and cell function experiments

FAM225B has been studied in other tumors or diseases and is associated with the development of nasopharyngeal carcinoma, glioblastoma, etc. [12–15]. However, relatively few studies have been conducted in renal clear cell carcinoma, so FAM225B was selected as the target of the follow-up study, and the sequence design for the FAM225B siRNA was as follows:

Si1: ATGCTTTCTGCAAAGAATAATAC
Si2: TGGTGCATGCATCTTTTCTGC
Si3: ATGTGTAATGAACATTTAAAATT

Using RNAi technology, transient transfection of siRNA was performed in renal cancer cell lines OS-RC-2 and 786-O using Lipofectamine 2000 (Invitrogen, USA), opti-MEM medium (Life Technologies, USA), and other reagents. After culturing the cells under optimal conditions, RNA was extracted from the cells, and RT-qPCR was performed to determine the knockdown efficiency. The above constructed cells were inoculated in 96-well plates, and the fluorescence values at 450 nm (OD value) of these two cells at 0 h, 24 h, 48 h, 72 h, and 96 h were measured using CCK-8 kit (Solarbio, Beijing, China), to compare the cell proliferation ability.

The logarithmic growth phase of the constructed transfected siRNA kidney cancer cells OS-RC-2 and 786-O were inoculated in six-well plates, and when the cells grew to 80–90% using a 1 ml pipette tip scratch, PBS (Phosphate Buffered Saline) was washed followed by the addition of serum-free culture medium, photographed by microscopy and recorded for 0 h for each subgroup, after which they were placed in a 37°C, 5% CO_2_ incubator. The 6-well plates were subsequently removed at 24 h to aspirate and discard the old medium, and PBS was gently blown to wash away the floating cells before adding the serum-free medium and continuing to take photographs to record the photographs of each subgroup.

Liquid Matrigel matrix gel (Corning, USA) was diluted 1:9 using RIPM-1640 medium without FBS (Fetal Bovine Serum) and double antibodies (Anti-penicillin and streptomycin). Afterward, 70 ul of diluted substrate gel was taken and spread flat in Transwell chambers in 24-well plates and left in the incubator for 1 hour to allow the substrate gel to solidify. After digestion of the cells, the cells were resuspended using 200 ul of RIPM-1640 medium without FBS according to the cell density, and the cell volume was adjusted to 5 × 10^4^ cells and placed in the upper chamber. After adding 600 ul of RIPM-1640 complete medium in the lower chamber, they were placed in the cell culture chamber. The culture was terminated at 48 h, the chambers were removed, and the medium was aspirated and washed twice with PBS. The Matrigel matrix gel and cells in the chambers were wiped with cotton swabs, and 600 ul of methanol was added to the lower chamber for 30 min. 600 ul of Giemsa stain was added to the lower chamber after aspiration of the methanol, and the cells were stained for 30 min.

### Statistical analysis

All data were statistically analyzed using R4.1.0 software (https://www.r-project.org/) and Kaplan-Meier survival analysis was used to analyze the differences between the two risk groups. Independent samples *t*-test was used to compare the differences between the groups and statistical difference was considered at *p* < 0.05.

### Data availability statement

The data supporting this study’s findings are available from the corresponding author upon reasonable request.

## RESULTS

### Acquisition of differentially expressed disulfidptosis-related lncRNAs

**Figure 1:**
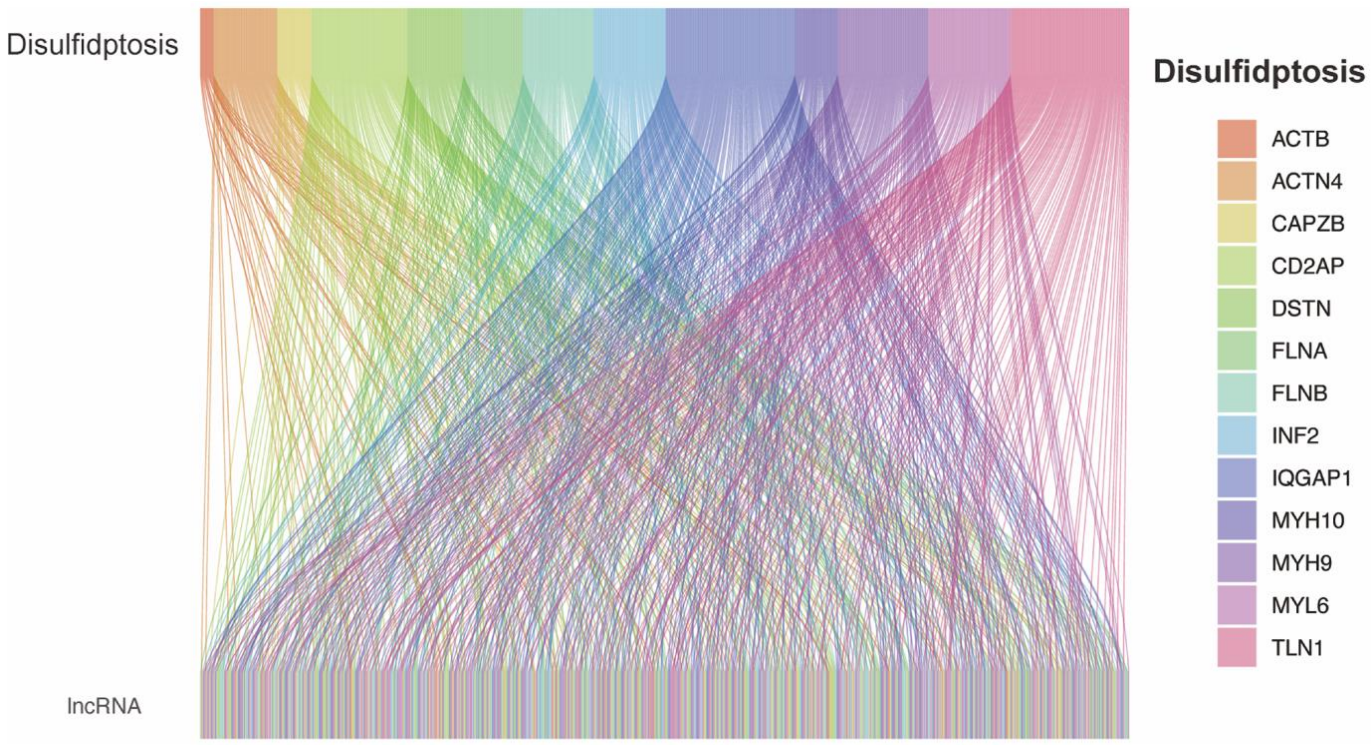
Co-expression of disulfidptosis-associated genes and lncRNAs.

**Figure 2:**
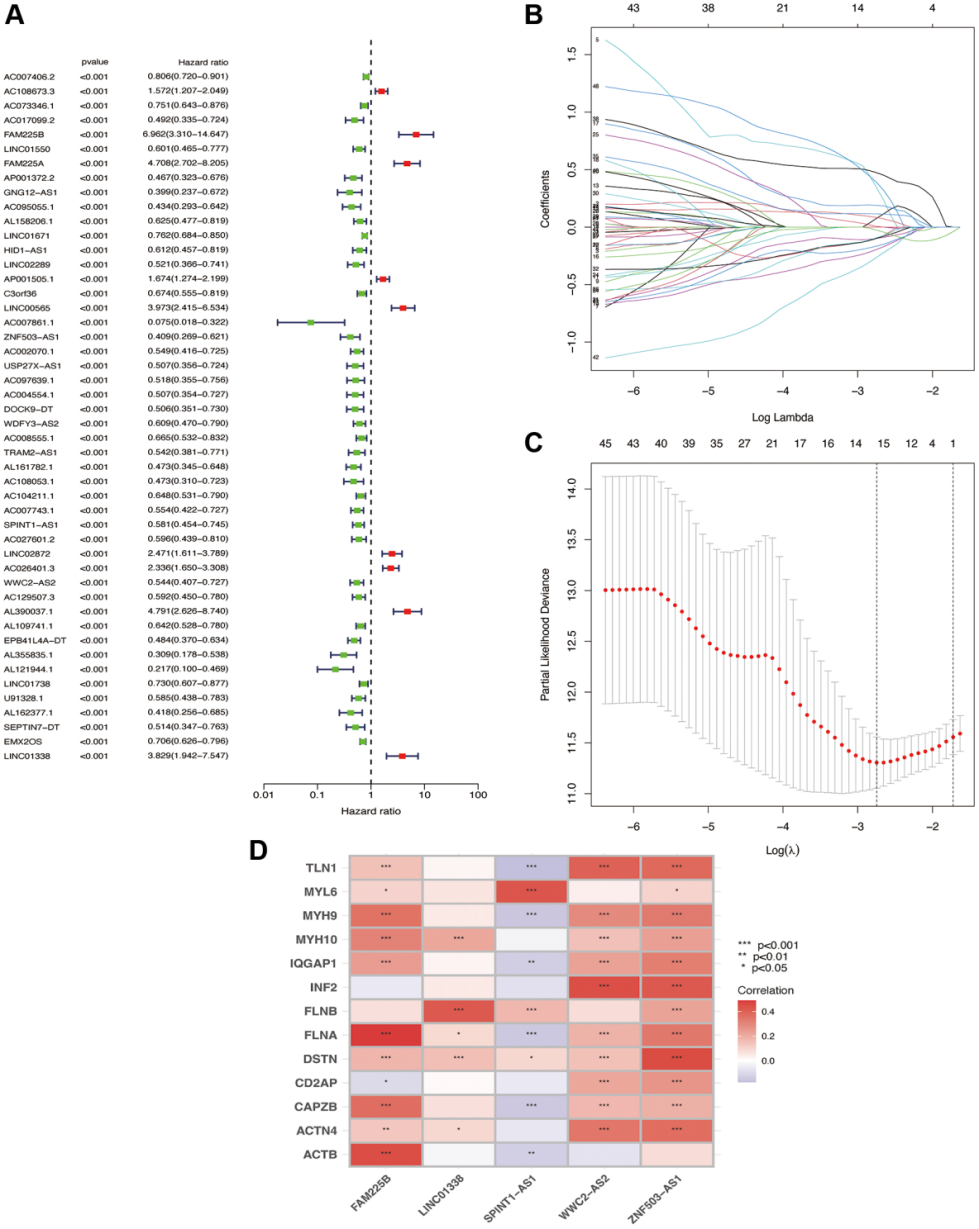
**Screening of lncRNA for independent prognosis.** One-way Cox regression analysis (**A**); Lasso regression analysis (**B**); λ values (**C**); correlation heat map (**D**).

From previous studies, we obtained 13 genes associated with Disulfideptosis. Through co-expression analysis, we identified 892 lncRNAs that showed co-expression relationships with these 13 Disulfideptosis-associated genes. The co-expression relationships are depicted in a Sankey diagram shown in Figure 1. Subsequently, we performed univariate Cox regression analysis on the aforementioned lncRNAs and identified 48 lncRNAs that were associated with the prognosis of renal clear cell carcinoma. The corresponding forest plot is shown in Figure 2A. After Lasso regression analysis and multifactorial Cox regression analysis (Figure 2B, 2C), 5 lncRNAs (FAM225B, ZNF503-AS1, SPINT1-AS1, WWC2-AS2, LINC01338) emerged as independent risk factors for the prognosis of renal clear cell carcinoma. The expression relationships of these five LncRNAs obtained with 13 genes related to Disulfidptosis were shown in Figure 2D.

### Development of a prognostic risk model

According to the formula Risk Score = (FAM225B × (1.21052350282848)) + (ZNF503-AS1 × (−0.413072607271419)) + (SPINT1-AS1 × (−0.348131526553965)) + (WWC2-AS2 × (−0.289726017312162)) + (LINC01338 × (1.00289905657691)). This indicates that FAM225B and LINC01338 are highly expressed in the high-risk group, while ZNF503-AS1, SPINT1-AS1, and WWC2-AS2 are lowly expressed in the high-risk group. We randomly divided the 533 samples in TCGA into two groups (train group with 267 cases and test group with 266 cases). The *t*-test results for the clinical data of both groups (Age, Gender, Grade, Stage, T, M, N) are presented in Supplementary Table 1. From the table, it is evident that there were no significant differences in all clinical data between the two randomly assigned groups (all *p* > 0.05), indicating satisfactory randomness of the two groups.

### Prognostic features of risk models

**Figure 3:**
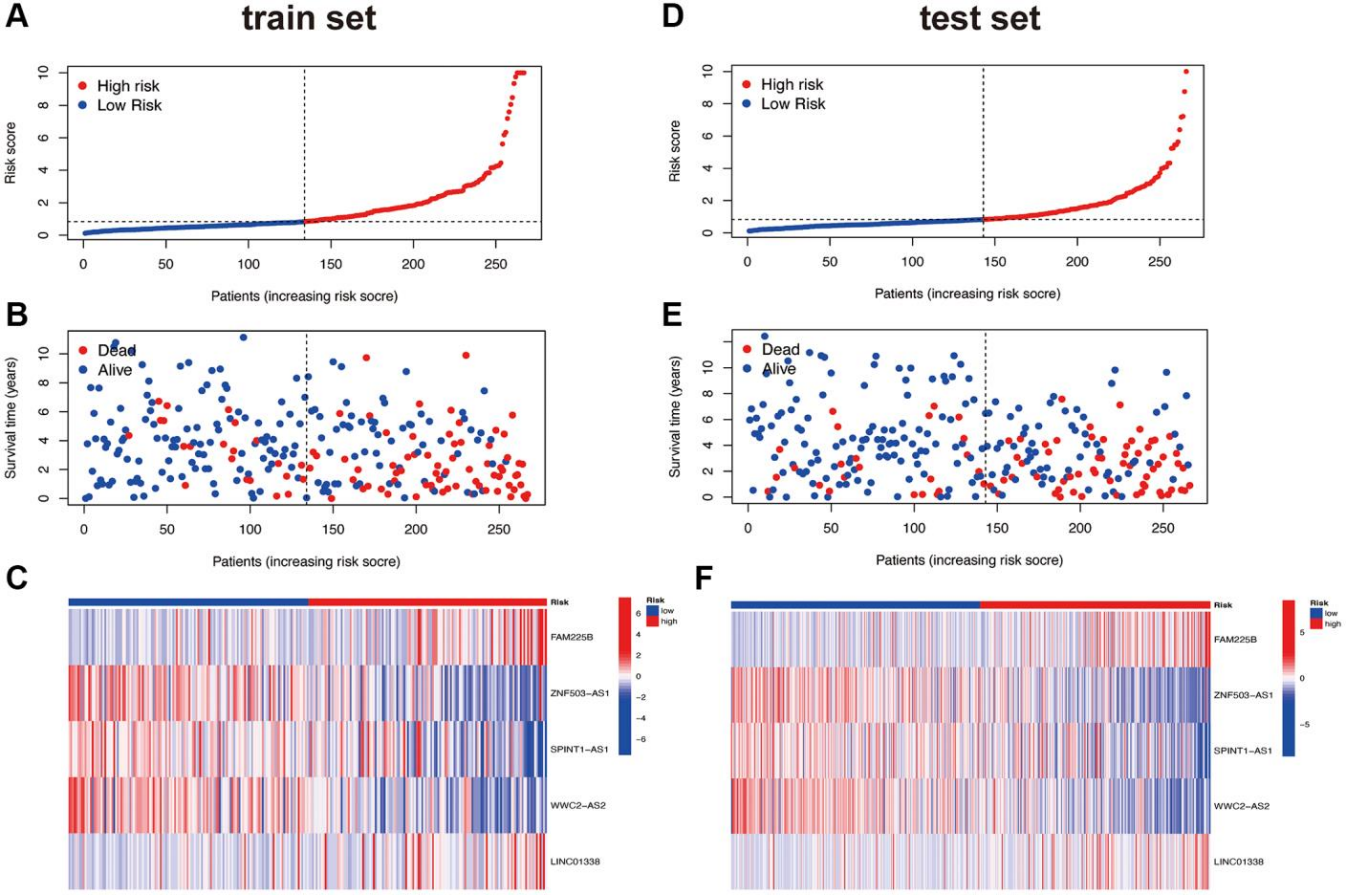
**Risk curve.** Train set (**A**–**C**); Test set (**D**–**F**).

Based on the risk scores obtained from the previous risk model, patients were divided into high and low-risk groups using the median method. The risk curves, survival status curves, and risk heatmaps of all patients are shown in Supplementary Figure 1A–1C. It is evident that our risk model effectively stratifies patients into high and low-risk groups. As the risk score increases, the number of patient deaths also increases, which aligns with our expectations. From the risk heatmap, it is also evident that FAM225B and LINC01338 are positively correlated with the risk score, while ZNF503-AS1, SPINT1-AS1, and WWC2-AS2 are negatively correlated with the risk score. This pattern is consistent in both the randomly assigned train and test groups, demonstrating the consistent and significant performance of the risk model. This internal validation of the risk model is shown in Figure 3A–3F.

### Clinical features and risk scores

**Figure 4:**
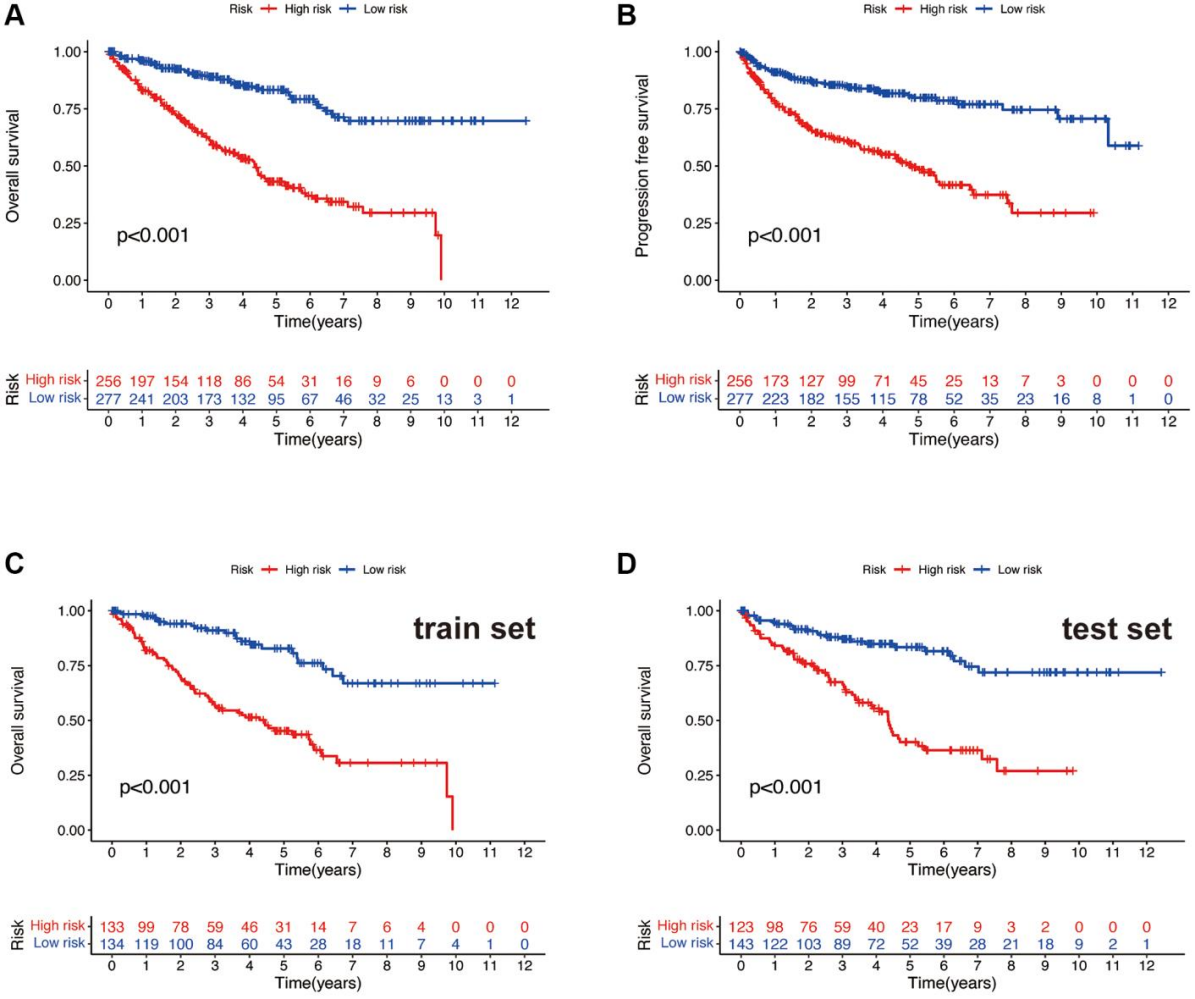
**Survival curve.** OS (**A**) and PFS (**B**) of the total sample. OS of the train set (**C**) and the test set (**D**).

After dividing all samples into high and low-risk groups based on the risk scores, Kaplan-Meier survival curves were used to compare the overall survival rates and progression-free survival rates in the high and low-risk groups of all samples. It is evident that both the overall survival time (Figure 4A) and progression-free survival time (Figure 4B) of the high-risk group are significantly lower than those of the low-risk group (all *p* < 0.001). To further validate the findings, in both the train and test sets, the high-risk group exhibited significantly lower overall survival time (Figure 4C, 4D) compared to the low-risk group (all *p* < 0.001). This indicates that the risk model can effectively distinguish patients with different survival outcomes, demonstrating its favorable predictive performance.

### Nomogram and clinical indicators

**Figure 5:**
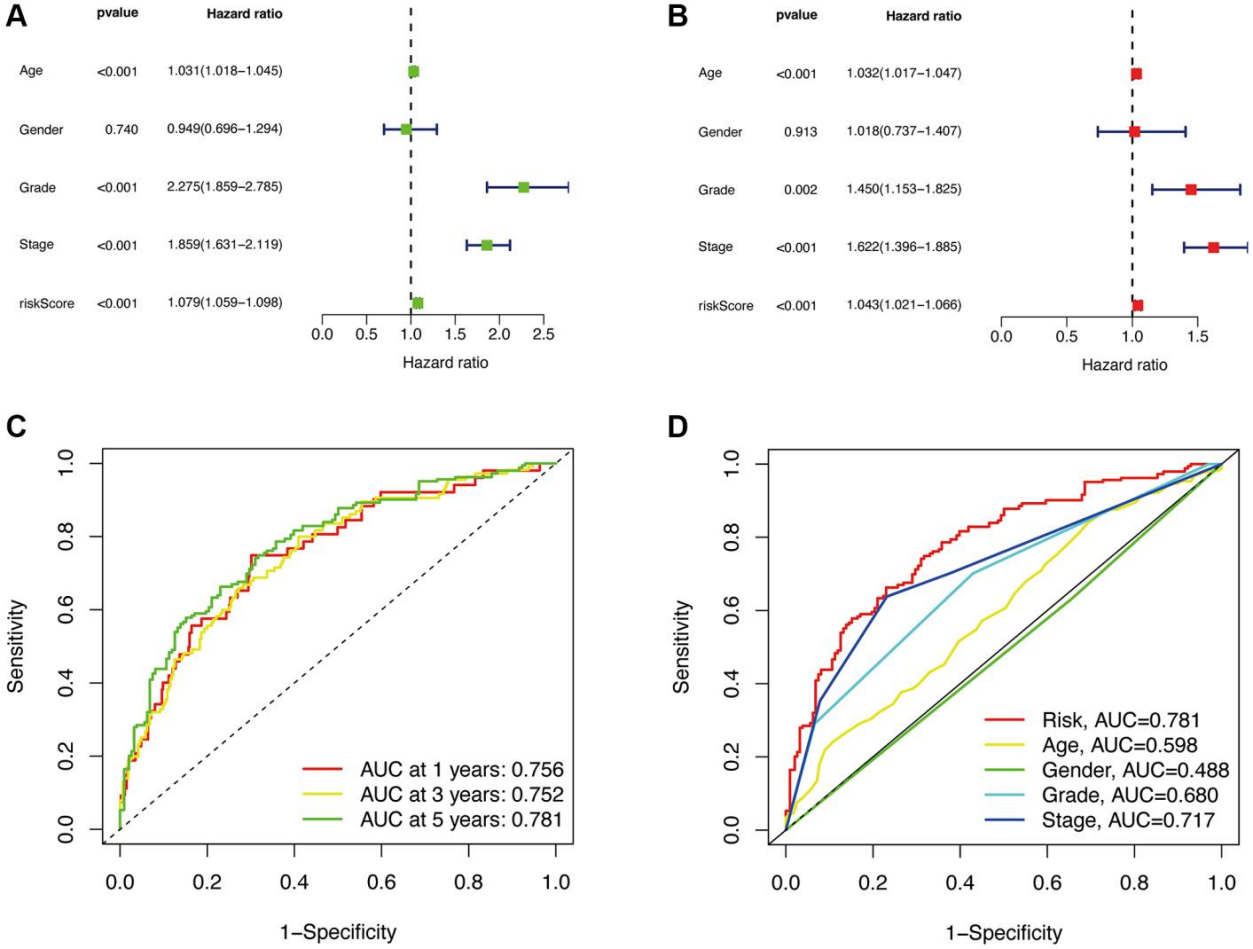
**Independent prognostic analysis.** Univariate analysis (**A**). Multivariate analysis (**B**). The ROC curve of the risk model for 1, 3, and 5 years (**C**). The 5-year ROC curve of the risk model in relation to other clinical characteristics (**D**).

To validate whether our constructed prognostic model is independent of other clinical characteristics (Age, Gender, Grade, Stage) as prognostic factors, we performed independent prognostic analysis. The results of univariate Cox regression analysis and multivariate Cox regression analysis showed that the risk score had a significant impact on prognosis, with *p* < 0.001 and hazard ratio of 1.079 (1.059–1.098) in univariate analysis, and *p* < 0.001 and hazard ratio of 1.043 (1.021-1.066) in multivariate analysis. These results are presented in Figure 5A, 5B, indicating that our constructed prognostic model is independent of other clinical characteristics and serves as an independent prognostic factor.

Subsequently, we calculated the AUC (Area Under the Curve) values of the prognostic model and plotted the ROC (Receiver Operating Characteristic) curve. The AUC values for 1, 3, and 5-year survival were 0.756, 0.752, and 0.781, respectively, indicating a high predictive value (Figure 5C). The AUC value (Figure 5D) for the 5-year survival (0.781) is higher compared to other clinical characteristics such as Age (0.598), Gender (0.488), Grade (0.680), and Stage (0.717). This indicates that our constructed prognostic model has a significant advantage over individual clinical characteristics in terms of predictive accuracy.

**Figure 6:**
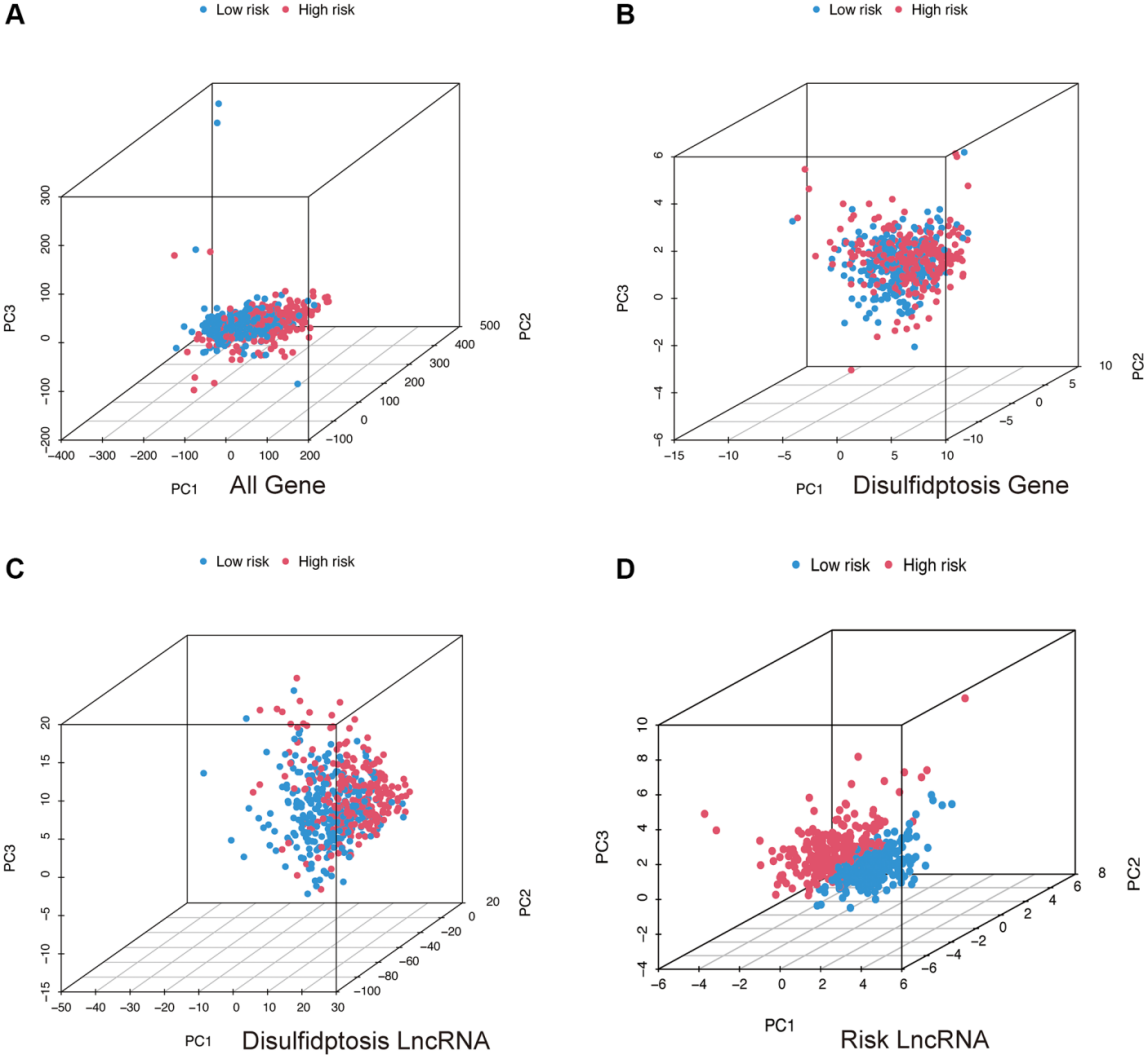
**PCA analysis.** All genes (**A**); Disulfidptosis genes (**B**); Disulfidptosis lncRNAs (**C**); Risk lncRNAs (**D**).

Through Principal Component Analysis (PCA), we can observe whether the lncRNAs involved in model construction can differentiate patients in the high and low-risk groups. From the three-dimensional visualization of the PCA results (Figure 6A–6D), it is evident that in Figure 6D, patients in the high-risk group are positioned in the upper left region of the three-dimensional plot, while patients in the low-risk group are positioned in the lower right region, with a clear boundary. Therefore, the lncRNAs involved in model construction can effectively distinguish patients in the high and low-risk groups.

**Figure 7:**
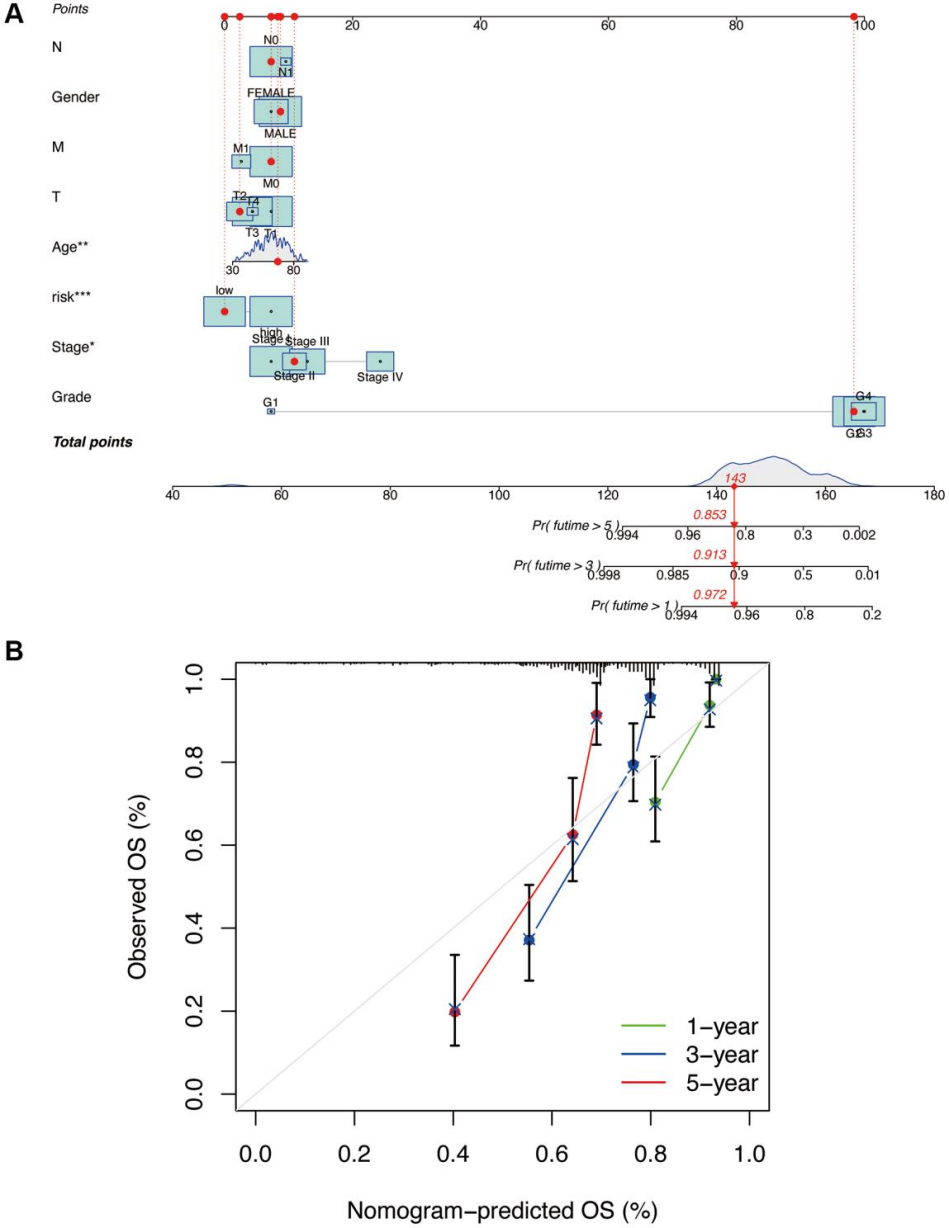
**Construction and validation of nomogram column line plots constructed from risk models.** A nomogram integrates risk scores and clinical characteristics to predict overall survival at 1, 3, and 5 years (**A**). Calibration curves for overall survival at 1, 3, and 5 years (**B**). ^*^p < 0.05, ^**^p < 0.01, ^***^p < 0.001.

Based on the risk model and related clinical data, we constructed a nomogram (Figure 7A). We also generated calibration curves for the nomogram at 1, 3, and 5-year intervals (Figure 7B). It can be observed that the three curves are relatively close to the reference line, indicating that the nomogram can accurately predict the survival period of patients.

**Figure 8:**
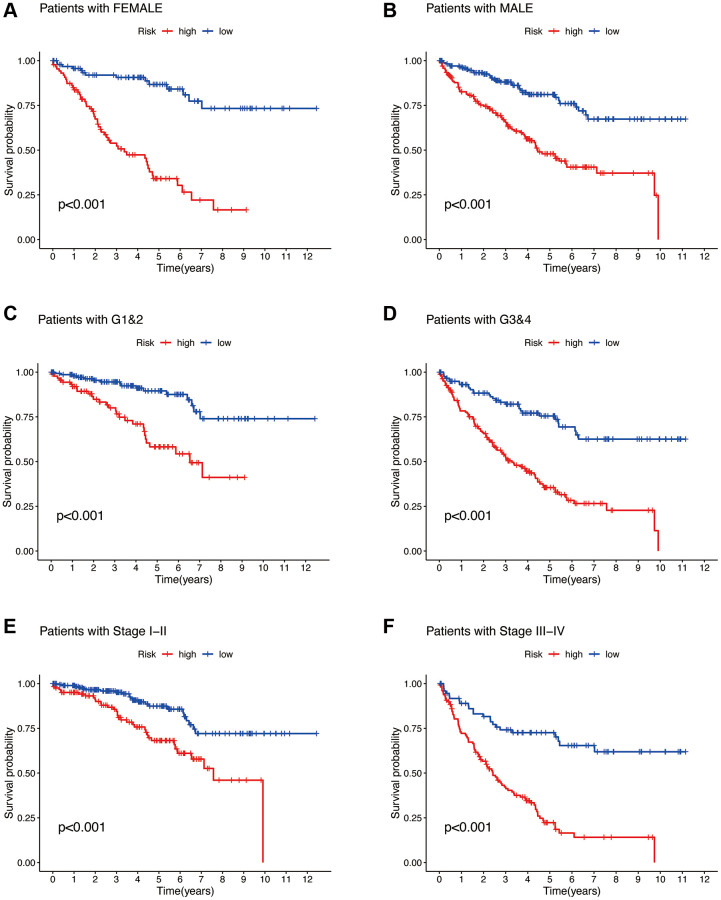
**Clinical subgroup analysis of the risk model.** Gender (**A**, **B**). Tumor grading (**C**, **D**). Tumor staging (**E**, **F**).

To investigate whether the risk model can be applied to patients in different clinical subgroups, we selected gender, Grade stage, and Stage classification as the clinical subgroups. From Figure 8A–8F, it is evident that our constructed risk model exhibits significant differences in survival probabilities between the high and low-risk groups in the FEMALE and MALE subgroups (Figure 8A, 8B), G1&2 and G3&4 subgroups (Figure 8C, 8D), and Stage I–II and Stage III–IV subgroups (Figure 8E, 8F) (all *p* < 0.001). This indicates that the risk model demonstrates excellent predictive performance in patients from different clinical subgroups as well.

### Functional and pathway enrichment analysis in the risk model

**Figure 9:**
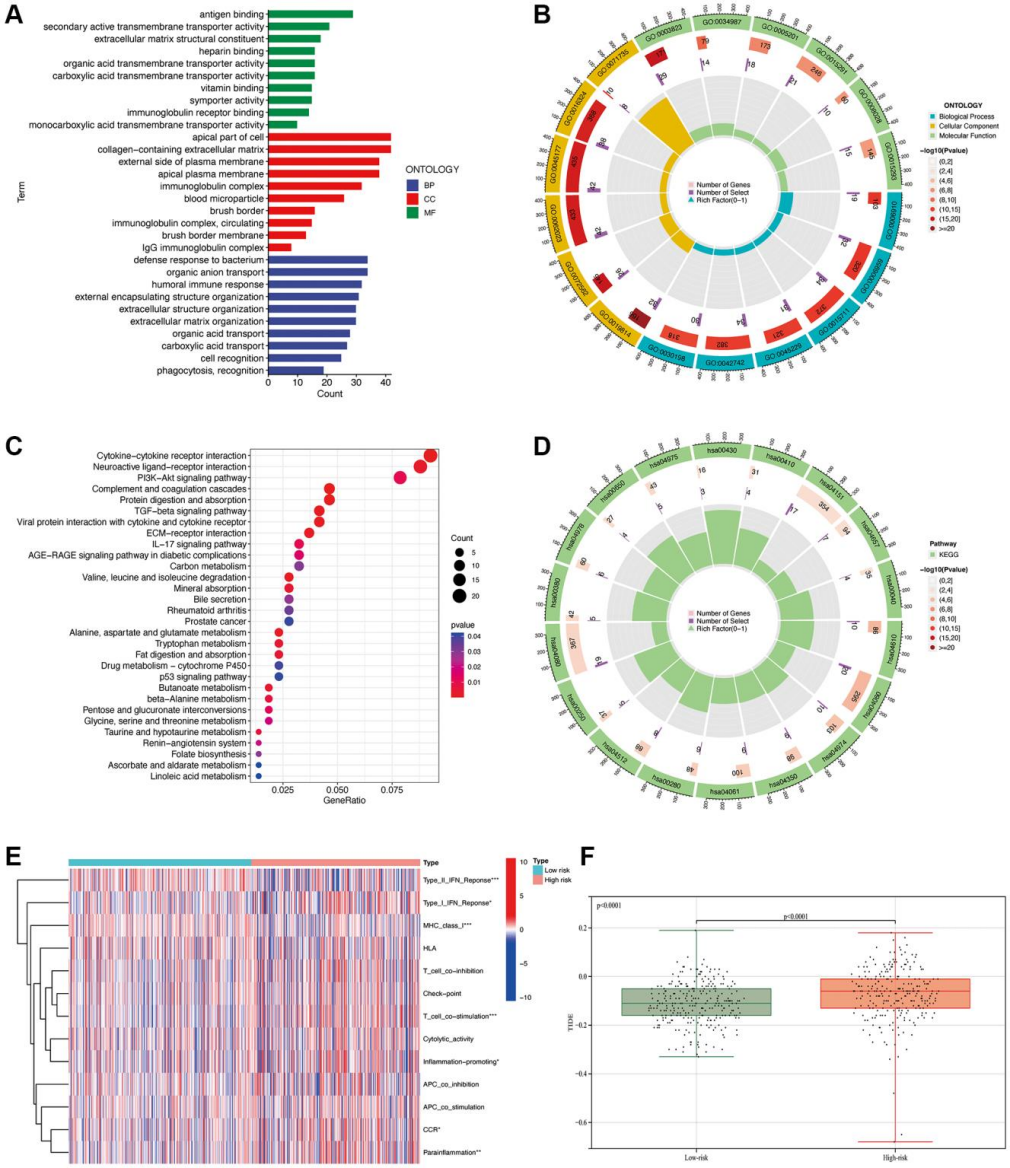
**Functional enrichment analysis.** GO analysis (**A**, **B**). KEGG pathway analysis (**C**, **D**). GSEA analysis (**E**). Comparison of TIDE values between high and low-risk groups (**F**). ^*^p < 0.05, ^**^p < 0.01, ^***^p < 0.001.

The bar plot of the GO analysis (Figure 9A) reveals that in terms of Molecular Function (MF), the main focuses are on antigen binding and secondary active transmembrane transponder activity. In Cellular Component (CC), the main focuses are on the apical part of the cell and the collagen-containing extracellular matrix. In Biological Process (BP), the main focuses are on the defense response to bacterium and organic anion transport. GO enrichment analysis bubble plot (Figure 9B): The outermost circle represents the GO enrichment IDs, the second circle represents the number of genes associated with each GO term, the third circle represents the number of differentially expressed genes enriched in each GO term, and the fourth circle represents the proportion of enriched genes. The redder the color, the more significant the enrichment of differentially expressed genes. From the figure, we can observe that the gene count enriched in the GO term “0071735” is the highest in proportion. For KEGG pathway analysis, the bar plot (Figure 9C) indicates that the highest enrichment and differential expression are primarily observed in pathways such as Cytokine-cytokine receptor interaction, Neuroactive ligand-receptor interaction, and PI3K-Akt signaling pathway. From the circular plot of the KEGG pathway analysis (Figure 9D), it is also evident that the results are primarily focused on hsa00430 and hsa04610. From the GSEA analysis (Figure 9E), it can be observed that there are significant differences between the high and low-risk groups in terms of Type_II_IFN_Reponse, Type_I_IFN_Reponse, MHC_class_I, T_cell_co_stimulation, Inflammation-promoting, CCR, and Parainflammation. The calculated TIDE values (Figure 9F) are significantly lower in the low-risk group compared to the high-risk group (*p* < 0.0001). This finding further indicates that our predictive model can effectively distinguish the immune escape capabilities of patients in the high and low-risk groups.

### Tumor mutational burden (TMB) analysis in the risk model

**Figure 10:**
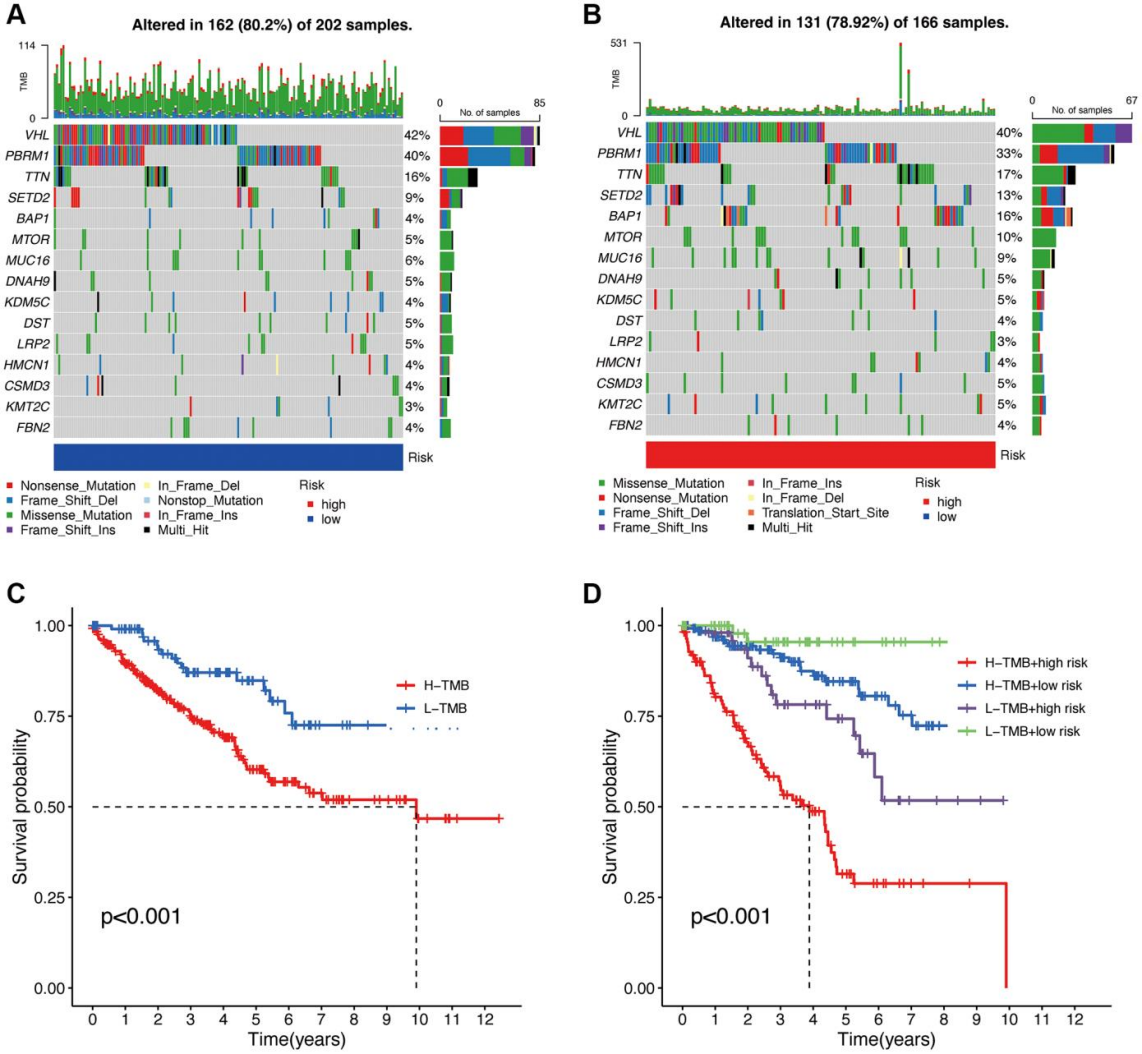
**Tumor mutational burden (TMB) analysis.** Waterfall plot of TMB in high and low-risk groups (**A**, **B**). Overall survival analysis of TMB (**C**). Survival analysis of TMB in different high and low-risk groups (**D**).

Based on the high and low TMB values, we divided the patients into high and low-risk groups (Figure 10A, 10B). The total mutation frequency in the low-risk group was 80.2%, while in the high-risk group, it was 78.92%, which is lower than that in the low-risk group. In the low-risk group, Nonsense Mutation was predominantly observed, while in the high-risk group, Missense Mutation was the major type. The top 15 genes with mutations, ranked by mutation frequency from high to low, are VHL, PBRM1, TTN, SETD2, BAP1, MTOR, MUC16, DNAH9, KDM5C, DST, LRP2, HMCN1, CSMD3, KMT2C, and FBN2. The KM curve of TMB in the high and low-risk groups shows that patients with low TMB have a higher probability of long-term survival. The combined analysis of TMB and the risk model score also reveals clear trends in the KM curve (Figure 10C). Patients with low TMB and low-risk scores tend to have better survival outcomes, while those with high TMB and high-risk scores have the poorest prognosis (Figure 10D).

### Results of *in vitro* cell function experiments

**Figure 11:**
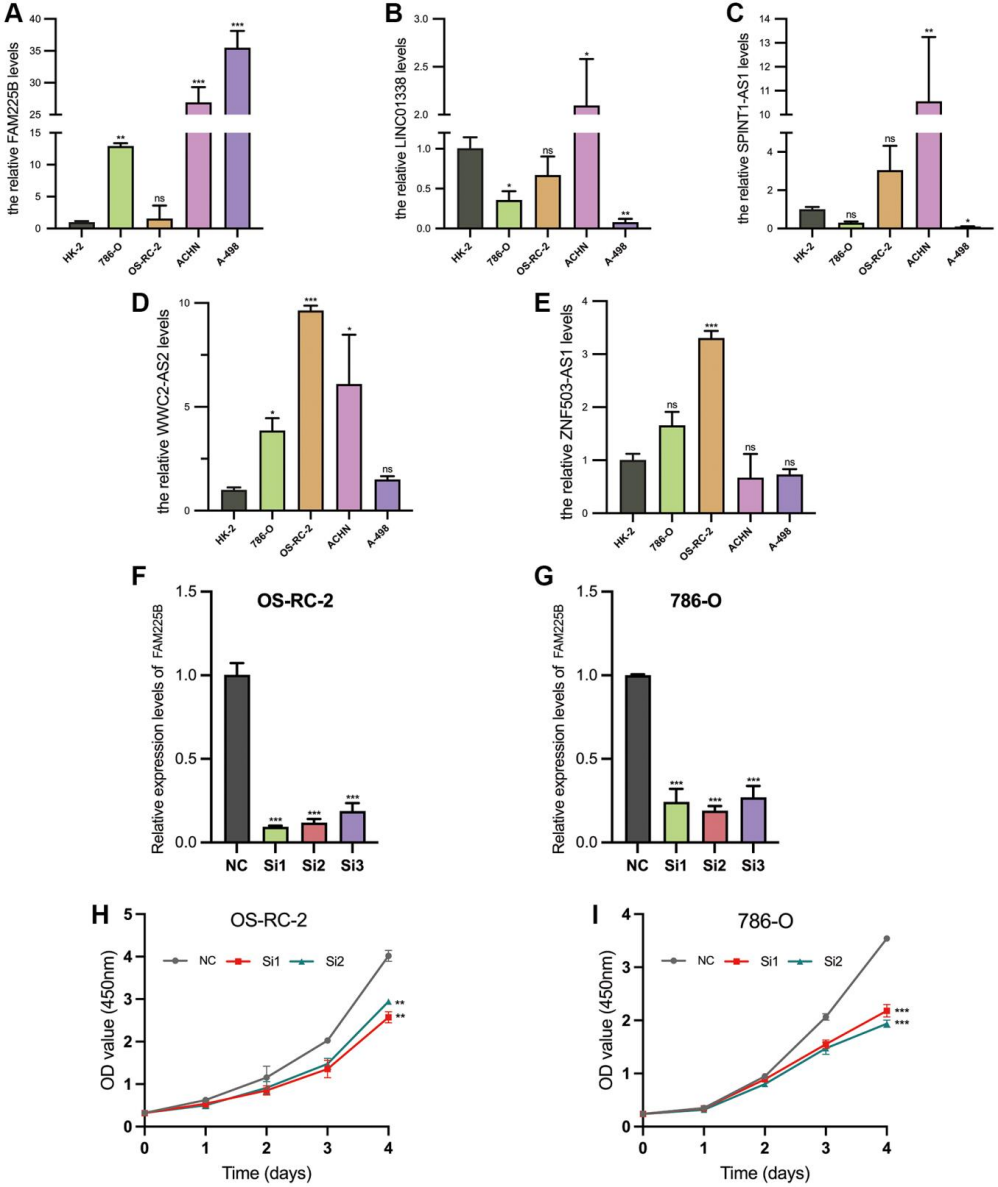
**Validation of 5 Disulfidptosis-associated lncRNAs.** Comparison of RNA expression in renal cancer cells (786-O, OS-RC-2, ACHN, A-498) and normal renal proximal tubular epithelial cells (HK-2) (**A**–**E**). Validation of knockdown potency of FAM225B (**F**, **G**). Validation of cellular value added after FAM225B knockdown (**H**, **I**). ^*^p < 0.05, ^**^p < 0.01, ^***^p < 0.001, ^ns^*p* > 0.05.

The validated expression levels of the five selected lncRNAs associated with Disulfideptosis (FAM225B, ZNF503-AS1, SPINT1-AS1, WWC2-AS2, LINC01338) in five cell lines (HK-2, 786-O, OS-RC-2, ACHN, A-498) are shown in Figure 11A–11E. From the results, it is evident that FAM225B and WWC2- AS2 are highly expressed in multiple tumor cell lines. The interference efficiency of the two renal tumor cell lines (OS-RC-2, 786-O) is depicted in Figure 11F, 11G. Subsequently, Si1 and Si2 were selected for further studies.

The OD values at 450 nm of OS-RC-2 and 786-O cells, measured by CCK-8 assay, after interference with Si1 and Si2 are shown in Figure 11H, 11I. It is evident that the proliferation of both cell lines is significantly reduced after interference.

**Figure 12:**
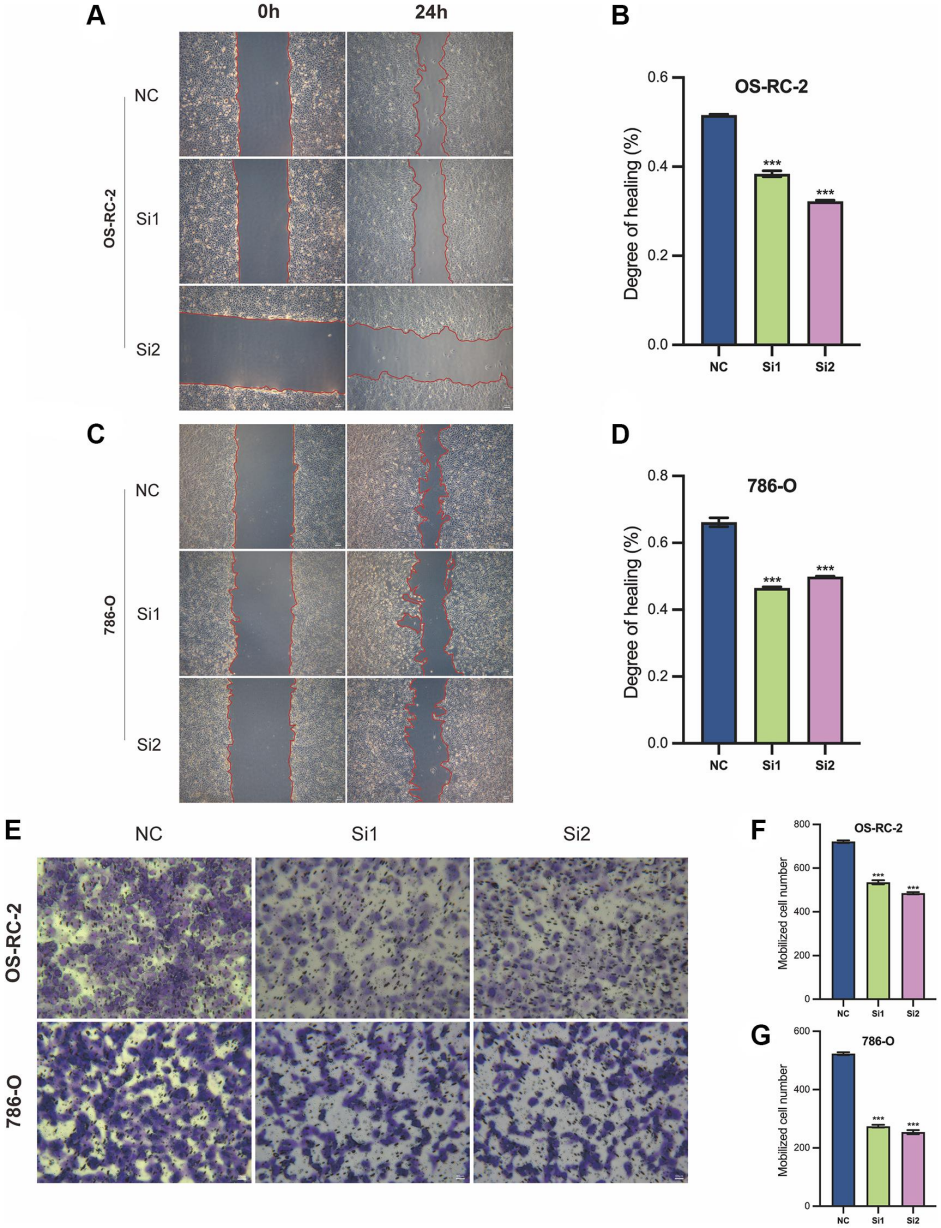
**Cellular function validation of FAM225B in two cell lines, 786-O, and OS-RC-2.** Wound healing assay (**A**–**D**). Cell invasion assay (**E**–**G**). ^***^p < 0.001.

In the *in vitro* cell migration assay, the wound healing capacity of OS-RC-2 cells after interference with Si1 and Si2 (Si1 and Si2 groups) is significantly lower compared to the control group (NC group) (Figure 12A, 12B). Similarly, this is also observed in 786-O cells (Figure 12C, 12D). These results indicate that the decreased expression of the LncRNA associated with Disulfideptosis (FAM225B) significantly affects the migration ability of both renal cancer cell lines.

In the cell invasion assay, the invasive capacity of OS-RC-2 cells after interference with Si1 and Si2 (Si1 and Si2 groups) is significantly lower compared to the control group (NC group) (Figure 12E, 12F). Similarly, this is also observed in 786-O cells (Figure 12G). These results indicate that the decreased expression of the lncRNA associated with Disulfidptosis (FAM225B) significantly affects the invasion ability of both renal cancer cell lines.

## DISCUSSION

Many cancer therapies aim to kill cancer cells by inducing apoptosis. However, cancer cells often find ways to evade apoptosis, leading to treatment resistance and disease relapse. Disulfideptosis, a recently discovered form of cell death, may open new avenues for cancer diagnosis and treatment strategies. Renal clear cell carcinoma is one of the most common malignancies globally, characterized by subtle early symptoms, high recurrence rates, and a propensity for metastasis, which have drawn clinical attention [16]. In recent years, the important role of long non-coding RNA (lncRNA) in the occurrence and development of various cancers has been increasingly recognized [17]. Especially certain lncRNAs that are associated with Disulfideptosis, their expression and their role in renal clear cell carcinoma have not been extensively studied. Therefore, their performance and significance in renal clear cell carcinoma warrant further investigation.

In this study, we developed a robust prediction model that demonstrated satisfactory performance. By incorporating relevant risk factors, we generated a predictive nomogram for clinical application. Our prediction model effectively distinguished between patients at high and low risk, both in the overall cohort and within subgroups, and we observed that patients classified as high risk had poorer prognoses. Subsequently, functional enrichment analysis was performed. Through this analysis, we found that the lncRNAs associated with Disulfidptosis were enriched in functions related to defense response to bacterium, apical part of cell, collagen-containing extracellular matrix, cytokine-cytokine receptor interaction, and other immune-related processes. This suggests their potential as immunotherapeutic targets in renal clear cell carcinoma. In the immune cell enrichment analysis, we found that the majority of immune cells were enriched in the high-risk group, including Type_I_IFN_Response, T_cell_co-stimulation, Parainflammation, etc., and there were significant differences between the high-risk and low-risk groups (*p* < 0.01). According to previous studies [18], Type_I_IFN_Response and CD8(+) T cells play a significant role in immune therapy for breast cancer. Type_I_IFN_Response and CD8(+) T cells play a significant role in immune therapy for breast cancer. Similarly, T_cell_co-stimulation determines the functional outcome of T cell receptor (TCR) signaling and plays a critical role in T cell biology [19]. These findings collectively indicate a strong correlation between lncRNAs related to Disulfidptosis and the immune response.

The five lncRNAs (FAM225B, ZNF503-AS1, SPINT1-AS1, WWC2-AS2, LINC01338) were identified as having the independent predictive ability through single-factor Cox regression analysis, Lasso regression analysis, and multi-factor Cox regression analysis. FAM225B is associated with DNA methylation in human follicles [12]. Moreover, FAM225B is highly expressed in most tumors, Dai et al. found that the long non-coding RNA FAM225B promotes the proliferation and metastasis of nasopharyngeal carcinoma cells [13]. Additionally, FAM225B can serve as a prognostic biomarker for glioblastoma and may be a potential therapeutic target for glioblastoma treatment [14]. It can potentially be a therapeutic target for glioblastoma. For ZNF503-AS1, high levels of ZNF503-AS1 in plasma are associated with a high prevalence of diabetic retinopathy [20]. Moreover, ZNF503-AS1 can also serve as a potential biomarker for breast cancer and colorectal cancer [21, 22]. In previous studies, SPINT1-AS1 has been confirmed as a potential biomarker for renal clear cell carcinoma [23], Furthermore, it has also been identified as an independent prognostic indicator in pituitary adenoma, colorectal cancer, esophageal squamous cell carcinoma, and melanoma [24–27]. WWC2-AS2 has been identified as an immune-related lncRNA in cervical cancer [28]. LINC01338 is a member of N7-methylguanosine-related lncRNAs in bladder cancer [29], It can also be used to accurately predict the prognosis of bladder cancer patients, providing strong guidance for clinicians in developing better-individualized precision treatment strategies.

TIDE (http://tide.dfci.harvard.edu/) stands for tumor immune dysfunction and rejection. It is a computational framework for assessing the likelihood of tumor immune escape in the gene expression profile of tumor samples [30]. In our study, the TIDE values of samples in the high-risk group were significantly higher than those in the low-risk group (*p* < 0.0001), indicating a higher likelihood of immune escape in the tumor cells of the high-risk group. Moreover, in subsequent cell experiments, the knockdown of FAM225 resulted in significant reductions in proliferation, migration, and invasion abilities in Kidney tumor cells (786-O and OS-RC-2). Tumor Mutational Burden (TMB) is a measure of the number of acquired (non-inherited) genetic mutations in tumor cells. TMB is considered a biomarker that can predict the response of tumors to immunotherapy, particularly in predicting response to immune checkpoint inhibitors such as PD-1/PD-L1 inhibitors and CTLA-4 inhibitors [31, 32]. Our model predicts a smaller tumor mutational load in patients in the high-risk group and differs across subgroups, suggesting that different high- and low-risk groups may have different immune checkpoints.

Stress-induced disulfide-driven Disulfidptosis is a regulated form of cell death (RCD) [33], For example, ferroptosis is an iron-dependent form of regulated cell death (RCD) induced by lipid peroxidation [34]. Disulfidptosis primarily occurs in cancer cells with high expression of SLC7A11, which results from an inadequate supply of NADPH to support the reduction of cystine to cysteine, leading to disulfide stress. Recent studies have found that overexpression of SLC7A11 promotes tumor growth, in part because it inhibits iron apoptosis, a regulated form of cell death induced by excessive lipid peroxidation [35]. Some recent studies of SLC7A11 molecules bound to nanomaterials have also provided further insight into the fact that SLC7A11 molecules are closely related to iron death [36]. Identification and characterization of cell death mechanisms not only enhance our fundamental understanding of cellular homeostasis but also provide important insights for the treatment of various diseases, including cancer [37–39]. Although many questions remain to be investigated, the predictive power of the predictive model composed of its associated lncRNAs for renal clear cell carcinoma will be evident to all.

## CONCLUSION

We have constructed and validated a prognostic model based on lncRNAs associated with Disulfidptosis. This model includes the lncRNAs FAM225B, ZNF503-AS1, SPINT1-AS1, WWC2-AS2, and LINC01338. The predictive model serves as an independent prognostic factor for clear cell renal cell carcinoma (ccRCC), and it is a good predictor of the high and low risk of a patient’s prognosis and can differentiate between the size of the mutational load of tumor cells and immune escape ability in high and low risk patients. The predictive model provides new directions for personalized treatment strategies for patients with ccRCC.

## Supplementary Materials

Supplementary Figure 1

Supplementary Table 1

## References

[1] Raychaudhuri S. How can we kill cancer cells: Insights from the computational models of apoptosis. World J Clin Oncol. 2010; 1:24–8. 10.5306/wjco.v1.i1.24 21603307 PMC3095455

[2] Ganini C, Montanaro M, Scimeca M, Palmieri G, Anemona L, Concetti L, Melino G, Bove P, Amelio I, Candi E, Mauriello A. No Time to Die: How Kidney Cancer Evades Cell Death. Int J Mol Sci. 2022; 23:6198. 10.3390/ijms23116198 35682876 PMC9181490

[3] Liu X, Nie L, Zhang Y, Yan Y, Wang C, Colic M, Olszewski K, Horbath A, Chen X, Lei G, Mao C, Wu S, Zhuang L, et al. Actin cytoskeleton vulnerability to disulfide stress mediates disulfidptosis. Nat Cell Biol. 2023; 25:404–14. 10.1038/s41556-023-01091-2 36747082 PMC10027392

[4] Liu X, Olszewski K, Zhang Y, Lim EW, Shi J, Zhang X, Zhang J, Lee H, Koppula P, Lei G, Zhuang L, You MJ, Fang B, et al. Cystine transporter regulation of pentose phosphate pathway dependency and disulfide stress exposes a targetable metabolic vulnerability in cancer. Nat Cell Biol. 2020; 22:476–86. 10.1038/s41556-020-0496-x 32231310 PMC7194135

[5] Moujalled D, Strasser A, Liddell JR. Molecular mechanisms of cell death in neurological diseases. Cell Death Differ. 2021; 28:2029–44. 10.1038/s41418-021-00814-y 34099897 PMC8257776

[6] Elias EE, Lyons B, Muruve DA. Gasdermins and pyroptosis in the kidney. Nat Rev Nephrol. 2023; 19:337–50. 10.1038/s41581-022-00662-0 36596918

[7] Kreuzaler P, Watson CJ. Killing a cancer: what are the alternatives? Nat Rev Cancer. 2012; 12:411–24. 10.1038/nrc3264 22576162

[8] Cano Garcia C, Nimer N, Piccinelli ML, Tappero S, Panunzio A, Barletta F, Incesu RB, Tian Z, Saad F, Kapoor A, Briganti A, Terrone C, Shariat SF, et al. Differences in overall survival between clear cell metastatic renal cell carcinoma patients versus population-based controls according to race/ethnicity in the United States. Ann Epidemiol. 2023; 79:65–70. 10.1016/j.annepidem.2023.01.003 36640918

[9] Siegel RL, Miller KD, Wagle NS, Jemal A. Cancer statistics, 2023. CA Cancer J Clin. 2023; 73:17–48. 10.3322/caac.21763 36633525

[10] Sung H, Ferlay J, Siegel RL, Laversanne M, Soerjomataram I, Jemal A, Bray F. Global Cancer Statistics 2020: GLOBOCAN Estimates of Incidence and Mortality Worldwide for 36 Cancers in 185 Countries. CA Cancer J Clin. 2021; 71:209–49. 10.3322/caac.21660 33538338

[11] Bukavina L, Bensalah K, Bray F, Carlo M, Challacombe B, Karam JA, Kassouf W, Mitchell T, Montironi R, O'Brien T, Panebianco V, Scelo G, Shuch B, et al. Epidemiology of Renal Cell Carcinoma: 2022 Update. Eur Urol. 2022; 82:529–42. 10.1016/j.eururo.2022.08.019 36100483

[12] Fuchs Weizman N, Wyse BA, Montbriand J, Jahangiri S, Librach CL. Cannabis significantly alters DNA methylation of the human ovarian follicle in a concentration-dependent manner. Mol Hum Reprod. 2022; 28:gaac022. 10.1093/molehr/gaac022 35674367 PMC9247704

[13] Dai W, Shi Y, Hu W, Xu C. Long noncoding RNA FAM225B facilitates proliferation and metastasis of nasopharyngeal carcinoma cells by regulating miR-613/CCND2 axis. Bosn J Basic Med Sci. 2022; 22:77–86. 10.17305/bjbms.2021.5691 34255617 PMC8860311

[14] Li J, Zhang Q, Ge P, Zeng C, Lin F, Wang W, Zhao J. *FAM225B* Is a Prognostic lncRNA for Patients with Recurrent Glioblastoma. Dis Markers. 2020; 2020:8888085. 10.1155/2020/8888085 33299501 PMC7704151

[15] Ma X, Liu L. Knockdown of FAM225B inhibits the progression of the hypertrophic scar following glaucoma surgery by inhibiting autophagy. Mol Med Rep. 2021; 23:204. 10.3892/mmr.2021.11843 33495826 PMC7821338

[16] Erwin GS, Gürsoy G, Al-Abri R, Suriyaprakash A, Dolzhenko E, Zhu K, Hoerner CR, White SM, Ramirez L, Vadlakonda A, Vadlakonda A, von Kraut K, Park J, et al. Recurrent repeat expansions in human cancer genomes. Nature. 2023; 613:96–102. 10.1038/s41586-022-05515-1 36517591 PMC9812771

[17] Cheng R, Li F, Zhang M, Xia X, Wu J, Gao X, Zhou H, Zhang Z, Huang N, Yang X, Zhang Y, Shen S, Kang T, et al. A novel protein RASON encoded by a lncRNA controls oncogenic RAS signaling in KRAS mutant cancers. Cell Res. 2023; 33:30–45. 10.1038/s41422-022-00726-7 36241718 PMC9810732

[18] Wang Q, Bergholz JS, Ding L, Lin Z, Kabraji SK, Hughes ME, He X, Xie S, Jiang T, Wang W, Zoeller JJ, Kim HJ, Roberts TM, et al. STING agonism reprograms tumor-associated macrophages and overcomes resistance to PARP inhibition in BRCA1-deficient models of breast cancer. Nat Commun. 2022; 13:3022. 10.1038/s41467-022-30568-1 35641483 PMC9156717

[19] Chen L, Flies DB. Molecular mechanisms of T cell co-stimulation and co-inhibition. Nat Rev Immunol. 2013; 13:227–42. 10.1038/nri3405 23470321 PMC3786574

[20] Han T, Li W, Zhang H, Nie D. Involvement of long non-coding RNA ZNF503 antisense RNA 1 in diabetic retinopathy and its possible underlying mechanism. Bioengineered. 2022; 13:14057–65. 10.1080/21655979.2022.2062988 35734878 PMC9342252

[21] Li Z, Yu J, Lv C, Luo Z. Cancer-associated fibroblasts-derived lncRNA signature as a putative biomarker in breast cancer. Front Oncol. 2022; 12:1028664. 10.3389/fonc.2022.1028664 36408190 PMC9667072

[22] Yin T, Zhao D, Yao S. Identification of a Genome Instability-Associated LncRNA Signature for Prognosis Prediction in Colon Cancer. Front Genet. 2021; 12:679150. 10.3389/fgene.2021.679150 34163531 PMC8215581

[23] Qi-Dong X, Yang X, Lu JL, Liu CQ, Sun JX, Li C, Wang SG. Development and Validation of a Nine-Redox-Related Long Noncoding RNA Signature in Renal Clear Cell Carcinoma. Oxid Med Cell Longev. 2020; 2020:6634247. 10.1155/2020/6634247 33425212 PMC7781722

[24] Yin H, Zheng X, Tang X, Zang Z, Li B, He S, Shen R, Yang H, Li S. Potential biomarkers and lncRNA-mRNA regulatory networks in invasive growth hormone-secreting pituitary adenomas. J Endocrinol Invest. 2021; 44:1947–59. 10.1007/s40618-021-01510-x 33559847

[25] Li C, Li W, Zhang Y, Zhang X, Liu T, Zhang Y, Yang Y, Wang L, Pan H, Ji J, Wang C. Increased expression of antisense lncRNA *SPINT1-AS1* predicts a poor prognosis in colorectal cancer and is negatively correlated with its sense transcript. Onco Targets Ther. 2018; 11:3969–78. 10.2147/OTT.S163883 30022840 PMC6044340

[26] Shen FF, Pan Y, Yang HJ, Li JK, Zhao F, Su JF, Li YY, Tian LQ, Yu PT, Cao YT, Zhang YW, Zhou FY. Decreased expression of SPINT1-AS1 and SPINT1 mRNA might be independent unfavorable prognostic indicators in esophageal squamous cell carcinoma. Onco Targets Ther. 2019; 12:4755–63. 10.2147/OTT.S206448 31417276 PMC6591775

[27] Li FW, Luo SK. Identification and Construction of a Predictive Immune-Related lncRNA Signature Model for Melanoma. Int J Gen Med. 2021; 14:9227–35. 10.2147/IJGM.S340025 34880662 PMC8647169

[28] Ye J, Chen X, Lu W. Identification and Experimental Validation of Immune-Associate lncRNAs for Predicting Prognosis in Cervical Cancer. Onco Targets Ther. 2021; 14:4721–34. 10.2147/OTT.S322998 34526775 PMC8435534

[29] Ren L, Yang X, Liu J, Wang W, Liu Z, Lin Q, Huang B, Pan J, Mao X. An innovative model based on N7-methylguanosine-related lncRNAs for forecasting prognosis and tumor immune landscape in bladder cancer. Cancer Cell Int. 2023; 23:85. 10.1186/s12935-023-02933-7 37158958 PMC10165842

[30] Chen X, Chen H, He D, Cheng Y, Zhu Y, Xiao M, Lan H, Wang Z, Cao K. Analysis of Tumor Microenvironment Characteristics in Bladder Cancer: Implications for Immune Checkpoint Inhibitor Therapy. Front Immunol. 2021; 12:672158. 10.3389/fimmu.2021.672158 33936117 PMC8082152

[31] Rakaee M, Adib E, Ricciuti B, Sholl LM, Shi W, Alessi JV, Cortellini A, Fulgenzi CAM, Viola P, Pinato DJ, Hashemi S, Bahce I, Houda I, et al. Association of Machine Learning-Based Assessment of Tumor-Infiltrating Lymphocytes on Standard Histologic Images With Outcomes of Immunotherapy in Patients With NSCLC. JAMA Oncol. 2023; 9:51–60. 10.1001/jamaoncol.2022.4933 36394839 PMC9673028

[32] Li S, Yu W, Xie F, Luo H, Liu Z, Lv W, Shi D, Yu D, Gao P, Chen C, Wei M, Zhou W, Wang J, et al. Neoadjuvant therapy with immune checkpoint blockade, antiangiogenesis, and chemotherapy for locally advanced gastric cancer. Nat Commun. 2023; 14:8. 10.1038/s41467-022-35431-x 36596787 PMC9810618

[33] Qi X, Li Q, Che X, Wang Q, Wu G. Application of Regulatory Cell Death in Cancer: Based on Targeted Therapy and Immunotherapy. Front Immunol. 2022; 13:837293. 10.3389/fimmu.2022.837293 35359956 PMC8960167

[34] Dixon SJ, Lemberg KM, Lamprecht MR, Skouta R, Zaitsev EM, Gleason CE, Patel DN, Bauer AJ, Cantley AM, Yang WS, Morrison B 3rd, Stockwell BR. Ferroptosis: an iron-dependent form of nonapoptotic cell death. Cell. 2012; 149:1060–72. 10.1016/j.cell.2012.03.042 22632970 PMC3367386

[35] Koppula P, Zhuang L, Gan B. Cystine transporter SLC7A11/xCT in cancer: ferroptosis, nutrient dependency, and cancer therapy. Protein Cell. 2021; 12:599–620. 10.1007/s13238-020-00789-5 33000412 PMC8310547

[36] Yang C, Chen Z, Wei M, Hu S, Cai M, Wang N, Guan Y, Li F, Ding Q, Ling D. A self-amplified ferroptosis nanoagent that inhibits the tumor upstream glutathione synthesis to reverse cancer chemoresistance. J Control Release. 2023; 357:20–30. 10.1016/j.jconrel.2023.03.030 36940774

[37] Li D, Shi Z, Liu X, Jin S, Chen P, Zhang Y, Chen G, Fan X, Yang J, Lin H. Identification and development of a novel risk model based on cuproptosis-associated RNA methylation regulators for predicting prognosis and characterizing immune status in hepatocellular carcinoma. Hepatol Int. 2023; 17:112–30. 10.1007/s12072-022-10460-2 36598701

[38] Consoli V, Fallica AN, Sorrenti V, Pittalà V, Vanella L. Novel Insights on Ferroptosis Modulation as Potential Strategy for Cancer Treatment: When Nature Kills. Antioxid Redox Signal. 2023. [Epub ahead of print]. 10.1089/ars.2022.0179 37132605 PMC10824235

[39] Zhang B, Wang Q, Zhang T, Zheng Z, Lin Z, Zhou S, Zheng D, Chen Z, Zheng S, Zhang Y, Lin X, Dong R, Chen J, et al. Identification and validation of a novel cuproptosis-related gene signature in multiple myeloma. Front Cell Dev Biol. 2023; 11:1159355. 10.3389/fcell.2023.1159355 37152283 PMC10157051

