# Supplementary material for: Disulfideptosis-associated lncRNAs reveal features of prognostic, immune escape, tumor mutation, and tumor malignant progression in renal clear cell carcinoma
Source: Aging (Albany NY). 2024 Feb 8;16(4):3280–301. doi: 10.18632/aging.205534 (PMC10929831; doi:10.18632/aging.205534)
Supplement: Supplementary Figure 1 [file aging-16-205534-s001.pdf]

SUPPLEMENTARY FIGURE

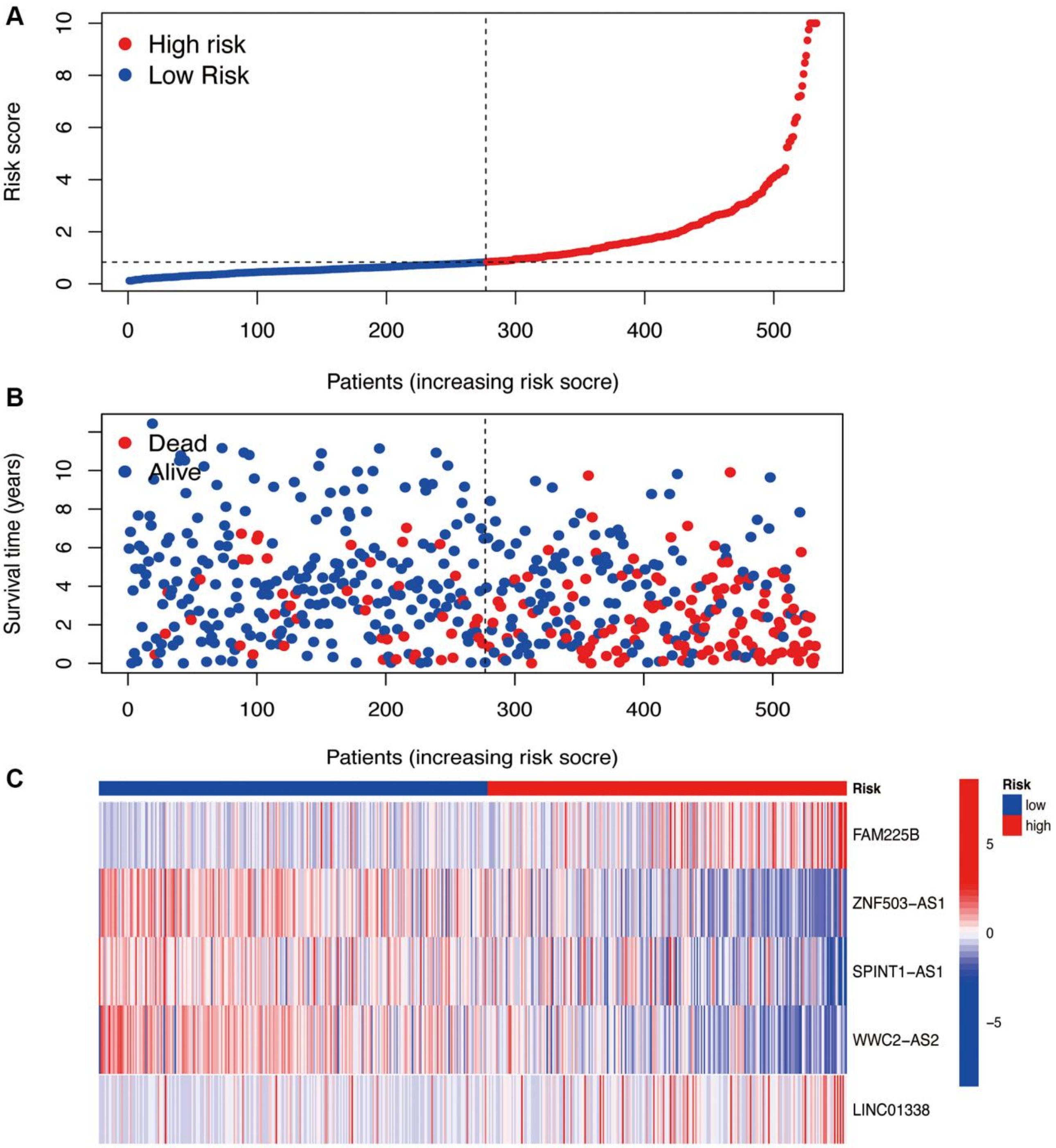

Supplementary Figure 1. Risk curves for all patients (**A**), survival curves (**B**), and risk heat map (**C**).
