# Supplementary material for: Disulfideptosis-associated lncRNAs reveal features of prognostic, immune escape, tumor mutation, and tumor malignant progression in renal clear cell carcinoma
Source: Aging (Albany NY). 2024 Feb 8;16(4):3280–301. doi: 10.18632/aging.205534 (PMC10929831; doi:10.18632/aging.205534)
Supplement: Supplementary Table 1 [file aging-16-205534-s002.pdf]

SUPPLEMENTARY TABLE

Supplementary Table 1. Results of *t*-test for clinical data of train and test sets.

Covariates	Type	Total	Test	Train	*P*-value
**Age**	≤65	349 (65.48%)	177 (66.54%)	172 (64.42%)	**0.6715**
	>65	184 (34.52%)	89 (33.46%)	95 (35.58%)	
**Gender**	Female	188 (35.27%)	91 (34.21%)	97 (36.33%)	**0.6735**
	Male	345 (64.73%)	175 (65.79%)	170 (63.67%)	
**Grade**	G1	14 (2.63%)	7 (2.63%)	7 (2.62%)	**0.4156**
	G2	229 (42.96%)	114 (42.86%)	115 (43.07%)	
	G3	206 (38.65%)	110 (41.35%)	96 (35.96%)	
	G4	76 (14.26%)	32 (12.03%)	44 (16.48%)	
	Unknown	8 (1.5%)	3 (1.13%)	5 (1.87%)	
**Stage**	Stage I	267 (50.09%)	136 (51.13%)	131 (49.06%)	**0.2856**
	Stage II	57 (10.69%)	25 (9.4%)	32 (11.99%)	
	Stage III	123 (23.08%)	68 (25.56%)	55 (20.6%)	
	Stage IV	83 (15.57%)	36 (13.53%)	47 (17.6%)	
	Unknown	3 (0.56%)	1 (0.38%)	2 (0.75%)	
**T**	T1	273 (51.22%)	138 (51.88%)	135 (50.56%)	**0.9687**
	T2	69 (12.95%)	33 (12.41%)	36 (13.48%)	
	T3	180 (33.77%)	90 (33.83%)	90 (33.71%)	
	T4	11 (2.06%)	5 (1.88%)	6 (2.25%)	
**M**	M0	422 (79.17%)	214 (80.45%)	208 (77.9%)	**0.2588**
	M1	79 (14.82%)	34 (12.78%)	45 (16.85%)	
	Unknown	32 (6%)	18 (6.77%)	14 (5.24%)	
N	N0	240 (45.03%)	118 (44.36%)	122 (45.69%)	**0.8718**
	N1	16 (3%)	7 (2.63%)	9 (3.37%)	
	Unknown	277 (51.97%)	141 (53.01%)	136 (50.94%)	
